# Pathway from interpersonal sensitivity to depression among Chinese college students during the COVID-19 campus lockdown

**DOI:** 10.1265/ehpm.22-00249

**Published:** 2023-06-07

**Authors:** Haibo Xu, Yifei Pei, Zheng Yong, Xin Liu, Wei Wang

**Affiliations:** 1 Center for Mental Health Education and Research, Xuzhou Medical University, Xuzhou, China; 2 School of Management, Xuzhou Medical University, Xuzhou, China; 3 School of Public Health, Xuzhou Medical University, Xuzhou, China; 4 Key Laboratory of Human Genetics and Environmental Medicine, Xuzhou Medical University, Xuzhou, China

**Keywords:** Interpersonal sensitivity, Anxiety, Psychological capital, Depression, Campus lockdown

## Abstract

**Background:**

Due to the continuous spread of the epidemic, some colleges and universities have implemented a campus lockdown management policy in China. In the context of the campus lockdown, this study aimed to explore whether anxiety mediated the association between interpersonal sensitivity and depression, and investigate whether psychological capital moderated the indirect or direct effect of mediation model.

**Methods:**

A total of 12945 undergrad students were recruited in China from April 10 to 19, 2022. These participants were asked to complete the online questionnaires measuring interpersonal sensitivity, anxiety, psychological capital, and depression. A moderated mediation model was examined by using PROCESS macro for SPSS 25.0, in which anxiety was a mediating variable, and psychological capital was a moderating variable.

**Results:**

Interpersonal sensitivity was positively associated with depression among Chinese college students (*r* = 0.47, *P* < 0.001). Anxiety partially mediated the association between interpersonal sensitivity and depression (indirect effect = 2.31, 95%CI [2.18, 2.44], accounting for 70% of the total effect). Moreover, the interaction effect of interpersonal sensitivity and psychological capital on anxiety (β = −0.04, *t* = −17.36, *P* < 0.001) and the interaction effect of anxiety and psychological capital on depression (β = 0.002, *t* = 1.99, *P* < 0.05) were statistically significant.

**Conclusions:**

The current study explained the mediation role of anxiety and the moderation role of psychological capital in the relation between interpersonal sensitivity and depression. The findings suggested that strict monitoring anxiety and promoting psychological capital may decrease the risk of depression among Chinese college students during the campus lockdown.

## Introduction

Due to the continuous spread of the epidemic, China continues its routine COVID-19 control measures in 2022 [1]. Some colleges and universities have implemented a campus lockdown management policy in China. However, the mental health of college students who are forced to stay on campus for extended periods due to campus closures may suffer. Previous researches have shown that many college students who lack the experience, analytical and predictive ability to deal with emergencies are more prone to depressive symptoms [2, 3]. During the period of closing campuses, the college students are not inclined to find ways to release pressure, which can also lead to depression states [4]. Consequently, the psychological condition of college students during the lockdown management of campus cannot be overlooked and must be monitored.

As one of the core concepts of interpersonal perception, interpersonal sensitivity is becoming an important variable in the field of social psychology. Interpersonal sensitivity is a personality tendency characterized by persistent fear of negative social evaluation, including separation anxiety, interpersonal consciousness, timidity and inner vulnerability [5]. Interpersonal sensitivity may result from negative core self-beliefs, a hot topic in depression research [6]. A number of existing cross-sectional surveys of Chinese college students have strongly proved the relationship. The studies indicated that college students who were overly sensitive to the behavior and feelings of others were vulnerable to developing depression [7–9]. Besides, the previous studies have shown that interpersonal sensitivity was a potent predictor of depression [10, 11]. Although interpersonal sensitivity may affect the depression of Chinese college students, the mediating role of pathway from interpersonal sensitivity to depression also needs to be explored in order to better clarify the internal influence mechanism.

In general, interpersonally sensitive individuals lack certain psychological meditations and set high goals for their daily actions. A study conducted in China among air force soldiers found that interpersonal sensitivity is connected with negative emotions [12]. Moreover, individuals having interpersonal sensitivity are more susceptible to concern about the interpersonal problems resulting from making mistakes [13]. College students who with higher interpersonal sensitivity are more likely to consider problems as threatening or unsolvable. When dealing with problems, they feel discouraged, as well as have lower self-efficacy, which further leads to problems related to anxiety [14]. On the other hand, anxiety and depression are closely related [15]. Researchers by using continuous measures of anxiety and depression found that anxiety positively predicted depression. This research suggested that anxiety may be a risk factor for depression [15]. Furthermore, clinical and epidemiological observations consistently indicated that anxiety can be considered a key factor in depression [16, 17]. In summary, these findings suggested that the relationship between interpersonal sensitivity and depression may be mediated by anxiety. If anxiety mediate the effects of interpersonal sensitivity on depression, interventions aimed at improving this mediator may have promising therapeutic potential.

Psychological capital is a personal resource, which is defined as “a positive mental state displayed by a person performs when growing up” [18]. As a deep motivation factor, psychological capital plays an active role in college students’ learning and daily life [19]. Some studies have shown that psychological capital can improve academic performance, relieve psychological pressure, and promote physical and mental health about college students [20–22]. Increasing researches in recent years have identified psychological capital as a moderator of negative emotions with significant predictive effects on depression [23–26]. In addition, existing clinical study found that psychological capital intervention has a significant effect on increasing psychological capital and alleviating depressive conditions [27]. Based on the broaden-and-built theory [25], psychological capital can help people make desirable emotional regulation strategies for negative emotions. A cross-sectional study performed among Chinese kindergarten teachers showed that psychological capital can significantly moderate the effects of emotional labor strategies on job burnout [28]. As mentioned earlier, psychological capital could be a desirable factor for college students to combat depression.

**Fig. 1:**
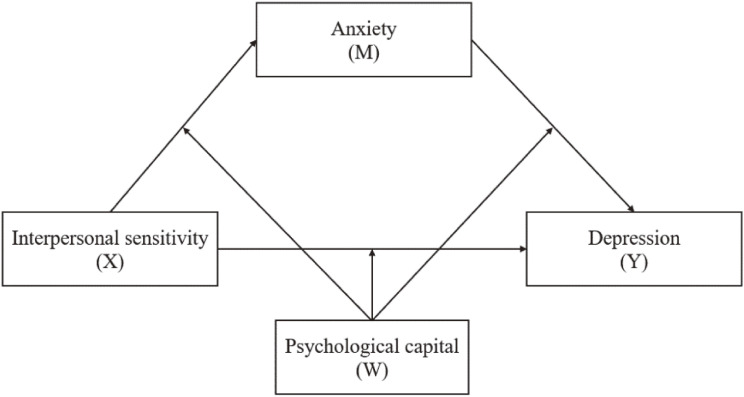
The hypothesis model of the relationships between interpersonal sensitivity, anxiety, psychological capital, and depression.

Although the impacts of interpersonal sensitivity, anxiety, and psychological capital with depression among Chinese college students were previously reported [7, 15, 26, 29], little is known about the potential mediating and moderating mechanisms underlying the associations in the context of the campus lockdown. This study aimed to explore whether anxiety mediated the association between interpersonal sensitivity and depression, and investigate whether psychological capital moderated the indirect or direct effect of mediation model in the context of the campus lockdown. We propose the following hypotheses according to the above literature: **Hypothesis 1**: Anxiety mediate the association between interpersonal sensitivity and depression among Chinese college students during the campus lockdown. **Hypothesis 2**: Psychological capital moderate the mediation model with interpersonal sensitivity as the predictor variable, anxiety as the mediator, and depression as the outcome. Figure 1 shows the proposed moderated mediation model.

## Methods

### Participants and procedure

This study used cross-sectional survey data from a university in eastern China based on all university students at school. The survey was conducted from April 10 to 19, 2022. Data were collected by the communication software WeChat or QQ (WeChat is a highly effective cross platform mobile phone software that provides instant messaging services for smart terminals for free. QQ is an Internet based instant messaging software launched by Tencent. They are all popular social media applications for Chinese college students.), and participants completed the Chinese questionnaire via scoring a QR code from WeChat or QQ. We excluded incomplete or nonconforming questionnaires (answer time less than 100 seconds, the same IP address) for quality control. Finally, a sample of 12,945 was used for analysis. All sample information was approved by the database owner for this study and was desensitized prior to the study to protect the personal privacy of the participants.

### Assessment

#### Basic characteristics

Information regarding the participants’ age, sex and original region were asked to understand the characteristics of the participants.

#### Interpersonal sensitivity

The interpersonal sensitivity data of the subjects were collected using the Symptom Checklist 90 (SCL-90) interpersonal sensitivity subscale. It was commonly adopted in experimental research on interpersonal sensitivity [12, 30]. There are nine items in the subscale, which are as follows (1) blame others and request them to be perfect, (2) feel uneasy and shy with the opposite sex, (3) feelings are easily hurt, (4) feel that others do not understand or sympathize with me, (5) feel that people are not friendly and do not like me, (6) feel inferior to others, (7) feel uncomfortable when people look at or talk about me, (8) feel nervous about others, and (9) feel uncomfortable when eating in public places. Respondents used a 5-point Likert scale (0 = no problem, 1 = very light, 2 = medium, 3 = heavy, 4 = very serious) to measure the extent to which they had experienced the listed symptoms. The nine scores were added as the interpersonal sensitivity score. Scores on this subscale range from 0–36. Higher scores were indicative of poorer interpersonal relationships in subjects. In this study, the Cronbach’s α for the scale was 0.91 in this study.

#### Anxiety

To evaluate the anxiety among the participants in the past two weeks, the Generalized Anxiety Disorder Scale [31] (GAD-7) was used. It involves seven items according to seven core symptoms. The specific items are as follows (1) feel nervous or anxious, (2) unable to stop or control concerns, (3) worry too much about all kinds of things, (4) it is hard to relax, (5) unable to sit still because of uneasiness, (6) become easily upset or irritable, and (7) feel afraid that something terrible will happen. Respondents report their conditions with a four-item Likert rating scale ranging from 0 (none) to 3 (always), and the total score ranges from 0 to 21. A higher total score indicated more symptoms with anxiety. In this study, the Cronbach’s α for the scale was 0.94.

#### Psychological capital

The Psychological Capital Questionnaire (PCQ-24), developed by Luthans et al., is a 24-item self-report scale including four dimensions: self-efficacy, hope, optimism, and tenacity [28]. Each of the four dimensions comprised six items, rated on a 6-point Likert-type scale (1 = strongly disagree, 6 = strongly agree). Higher values indicated higher levels of experienced psychological capital. The PCQ has been widely used, and demonstrates adequate reliability and validity in multiple samples [23, 28]. In this study, the Cronbach’s α for the scale was 0.96.

#### Depression

The depression of the participants was assessed using the Patient Health Questionnaire (PHQ-9) [32, 33], which consists of nine items. As follows: (1) have no interest or pleasure in doing anything, (2) feel depressed or hopeless, (3) difficult to fall asleep, hard to sleep well or sleep too much, (4) feel tired or listless, (5) loss of appetite or eat too much, (6) feel bad, or let yourself or your family down, (7) it is difficult to concentrate, such as reading newspapers or watching TV, (8) move or speak slowly to the extent that others can perceive, or just the opposite-you are fidgety or restless, and the situation of moving around is more serious than usual, and (9) it is better to die or hurt yourself in some way. Participants were asked to report the presence of nine problems in the last 2 weeks on a 4-point scale ranging from “nearly every day” (3 points) to “not at all” (0 points). The scores for depression severity were 5–9 for mild, 10–14 for moderate, and 15–19 moderately severe, 20–27 for severe. PHQ-9 has been widely used to assess depression in adolescents [34, 35]. In this study, the Cronbach’s α for the scale was 0.90.

### Statistical analysis

SPSS version 25.0 was used for all data analyses. The sociodemographic characteristics of the sample were presented using descriptive statistics. First, Pearson’s correlation analysis was used to investigate the links between interpersonal sensitivity, anxiety, psychological capital, and depression. Second, PROCESS v 3.4 macro for SPSS 25.0 was used to examine the potential mechanisms between interpersonal sensitivity, anxiety, psychological capital and depression, and the model 4 and 59 was selected to test the mediated and moderated mediation models [36]. Model 4 mediation analysis was used to judge if anxiety mediated the link between interpersonal sensitivity and depression. Finally, a moderated mediation modeling analysis (Model 59) was performed to investigate the moderated effect of psychological capital on the proposed mediation model. In the mediated and moderated mediation models, age, sex and original region were all considered covariates. Prior to the analysis, all variables in the moderated mediation model were centralized. In all statistical analyses, *P* < 0.05 was significant.

### Ethical approval and data collection

This study was a non-intervention study based on the sample database, which was approved by the Ethics Committee of Xuzhou Medical University. All research methods were performed in accordance with relevant guidelines of Declaration of Helsinki. All samples’ information was approved by the database owner for using in this study, and information’s desensitization was conducted before the study to protect the participants’ personal privacy. All participants in the questionnaire fulfilled the requirement of informed consent.

## Results

### Sociodemographic characteristics

Descriptive statistics were reported in Table 1. Of these 12945 college students, 9517 (73.5%) were between 19 and 22 years of age. The results showed that 57.3% of the participants were female, 56.4% were urban residents.

**Table 1:** Sociodemographic characteristics of participants (n = 12945)

**Variables**	**Classification**	**N**	**%**
**Age**			
	≤18	937	7.2
	19–22	9517	73.5
	≥23	2491	19.3
**Sex**			
	Male	5533	42.7
	Female	7412	57.3
**Original region**			
	Urban resident	7304	56.4
	Rural resident	5641	43.6

### Preliminary correlation analyses

**Fig. 2:**
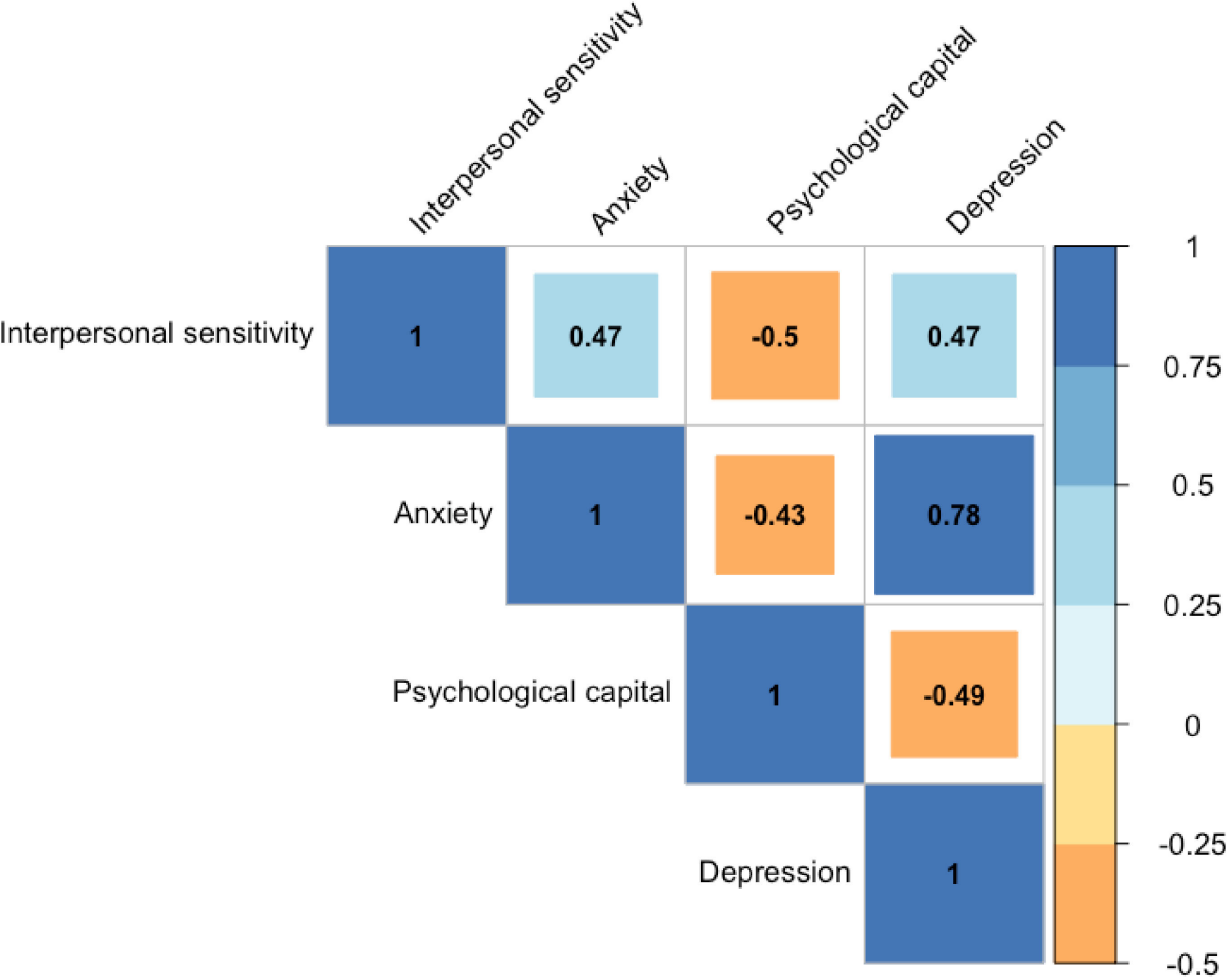
Pearson correlation matrix for all study variables (n = 12945), all correlations are significant at the *P* < 0.001 level.

Pearson’s correlations(r) between interpersonal sensitivity, anxiety, psychological capital, and depression were shown in Fig. 2. According to the results, interpersonal sensitivity was positively connected with both anxiety (r = 0.47, *P* < 0.001) and depression (r = 0.47, *P* < 0.001). Anxiety and depression were positively correlated (r = 0.78, *P* < 0.001). Nevertheless, interpersonal sensitivity (r = −0.50, *P* < 0.001), anxiety (r = −0.43, *P* < 0.001) and depression (r = −0.49, *P* < 0.001) were negatively associated with psychological capital.

### Mediation modeling analysis

**Fig. 3:**
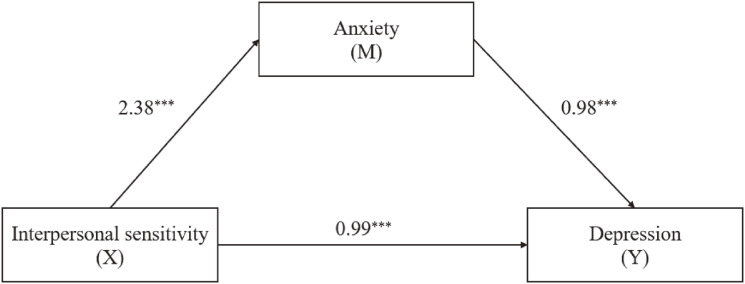
Mediated model for the whole sample (n = 12945), with standardized beta weights and significant level. Age, sex and original region were considered as covariates. ***P < 0.001

The results of the mediation modeling analysis indicated that interpersonal sensitivity was significantly positively associated with anxiety (β = 2.38, 95%CI [2.29, 2.46], *P* < 0.001) and depression (β = 0.99, 95%CI [0.90, 1.08], *P* < 0.001) in Table 2 and Fig. 3. Additionally, anxiety and depression had a significant positive relationship (β = 0.98, 95%CI [0.96, 0.99], *P* < 0.001). According to the results, the connection between interpersonal sensitivity and depression was shown to be partially mediated by anxiety (indirect effect = 2.31, 95%CI [2.18, 2.44], accounting for 70% of the total effect).

**Table 2:** Testing the mediation effect of interpersonal sensitivity on depression

**Predictors**	**B**	**SE**	***t***	***P***	**95%CI**
**Anxiety**					
Interpersonal sensitivity	2.38	0.04	53.87	<0.001	[2.29, 2.46]
[Model R = 0.47; R^2^ = 0.22; MSE = 10.13; F = 734.78; *P* < 0.001]					
**Depression**					
Anxiety	0.98	0.01	102.98	<0.001	[0.96, 0.99]
Interpersonal sensitivity (direct effect)	0.99	0.05	20.59	<0.001	[0.90, 1.08]
[Model R = 0.79; R^2^ = 0.62; MSE = 9.43; F = 3344.15; *P* < 0.001]					
**Total and indirect effects**
Total effect	3.30	0.06	54.68	<0.001	[3.19, 3.43]
Indirect effect	2.31	0.06	-	-	[2.18, 2.44]

**Note**: Covariates taken into account were age, sex and original region. The beta weights in the results were standardized.

### Moderated mediation modeling

**Fig. 4:**
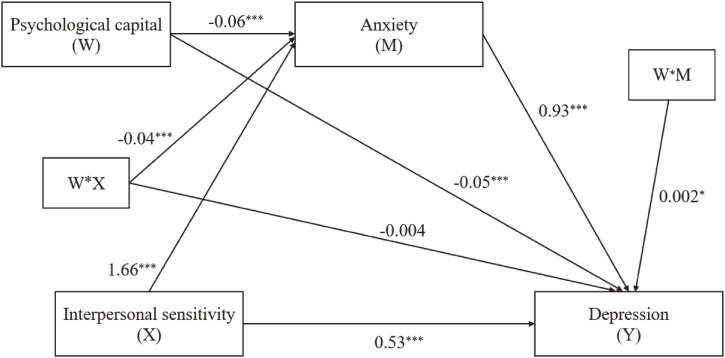
Statistical diagram of moderated mediation model in this research, with standardized beta weights. Covariates included age, sex and original region. **Note**: W*X is interactive term of psychological capital (W) and interpersonal sensitivity (X); W*M is interactive term of psychological capital (W) and anxiety (M). *P < 0.05, ***P < 0.001

The path results of the hypothesized moderated mediation model were shown in Fig. 4 and Table 3 summarized all of the results. The interaction effect of interpersonal sensitivity and psychological capital on anxiety (β = −0.04, t = −17.36, *P* < 0.001) and the interaction effect of anxiety and psychological capital on depression (β = 0.002, t = 1.99, *P* < 0.05) were both statistically significant. However, there was no significance of the interaction effect of interpersonal sensitivity and psychological capital on depression (β = −0.004, t = −1.52, *P* = 0.13).

**Table 3:** Testing the moderated mediation effect of interpersonal sensitivity on depression

**Predictors**	**B**	**SE**	***t***	***P***	**95%CI**
**Anxiety**					
Interpersonal sensitivity	1.66	0.05	34.07	<0.001	[1.56, 1.75]
Psychological capital	−0.06	0.002	−27.66	<0.001	[−0.07, −0.05]
Interpersonal sensitivity × Psychological capital	−0.04	0.002	−17.36	<0.001	[−0.05, −0.03]
[Model R = 0.54; R^2^ = 0.29; MSE = 9.20; F = 713.33; *P* < 0.001]					
**Depression**					
Interpersonal sensitivity	0.53	0.05	10.29	<0.001	[0.43, 0.63]
Anxiety	0.93	0.01	84.32	<0.001	[0.91, 0.95]
Psychological capital	−0.05	0.002	−23.90	<0.001	[−0.06, −0.04]
Interpersonal sensitivity × Psychological capital	−0.004	0.003	−1.52	0.13	[−0.009, 0.001]
Anxiety × Psychological capital	0.002	0.001	1.99	0.02	[0.001, 0.003]
[Model R = 0.80; R^2^ = 0.64; MSE = 8.94; F = 2276.28; *P* < 0.001]					

**Note**: Covariates taken into account were age, sex and original region. The beta weights in the model analysis were standardized.

The conditional indirect effects of interpersonal sensitivity on depression (through anxiety) were shown in Table 4 for incremental psychological capital levels ranging from low levels (i.e., 1 SD below the mean) to high levels (i.e., 1 SD above the mean). The indirect effect of anxiety in mediating the effect of interpersonal sensitivity on depression decreased from 2.08 to 0.97 when psychological capital increased from low levels (1 SD below the mean) to high levels (1 SD above the mean) (data were centralized). Simple slope tests showed that when psychological capital was at low (β = 2.08, 95%CI [1.91, 2.24]), moderate (β = 1.53, 95%CI [1.41, 1.67]), and high (β = 0.97, 95%CI [0.82, 1.13]) levels, the indirect effect of interpersonal sensitivity on depression through anxiety were significant.

**Table 4:** Conditional indirect effect of interpersonal sensitivity on depression at special level of psychological capital

**Mediator**	**Psychological capital**	**Effect ** **β**	**Boot ** **SE**	**Boot ** **LLCI**	**Boot ** **ULCI**
Anxiety	−16.73	2.08	0.08	1.91	2.24
	0.00	1.53	0.07	1.41	1.67
	16.73	0.97	0.08	0.82	1.13

**Note**: Data were centralized.

## Discussion

To our knowledge, this is the first study emphasizing the underlying mechanisms of interpersonal sensitivity, anxiety, psychological capital, and depression among Chinese college students during the campus lockdown. Our results showed that interpersonal sensitivity was positively associated with depression, anxiety partially mediated the link of interpersonal sensitivity–depression, and psychological capital moderated the indirect path between interpersonal sensitivity and depression. These findings may help researchers to better understand how interpersonal sensitivity affects depression among Chinese college students during the campus lockdown.

### The mediating role of anxiety

Our results showed that interpersonal sensitivity not only directly predicted depression but also indirectly affected depression through the mediating variable of anxiety. In terms of the relationship between interpersonal sensitivity and anxiety, the existing researches have demonstrated that interpersonal sensitivity was related to negative mental health outcomes, including PTSD, depression, and anxiety [37, 38]. Furthermore, a study investigated the interpersonal sensitivity of patients having social anxiety disorder, and found that the social anxiety disorder group had higher interpersonal sensitivity scores than the control group [39]. Under the campus lockdown management policy, college students were allowed to be active in the campus, which would strengthen the development of students’ introverted individual psychology. Young people with sensitive interpersonal relationships are more likely to be anxious during COVID-19 [40], which may be related to their sensitivity to social media reports. On the one hand, they are worried about the overall fear caused by insufficient protective measures to prevent COVID-19 infection; On the other hand, they fear that their headache, fatigue and tension are caused by COVID-19 infection. College students who with higher interpersonal sensitivity always spend more time with classmates and are more likely to tend to be overly concerned about possible negative feedback from others even over-react [41]. Over time, the repressed emotions cannot be transferred in time will lead to anxiety [14]. What’s more, anxious people cope with negative emotions by them, which leads to higher levels of depression later in life [15]. Besides, as mentioned in the Introduction section, anxiety may positively predict depression. Consequently, during the lockdown management of campus, we should pay attention to not only the symptoms of depression themselves, but also interpersonal sensitivity and anxiety.

### The moderating role of psychological capital

The present study found that psychological capital attenuated the link between interpersonal sensitivity and anxiety. Conservation of resources theory [42] proposed that valuable resources including material, power, and positive psychological factors [24, 43, 44] played a positive role in the process of the individual stress response. This theory provided a reasonable perspective to demonstrate that psychological capital might help people deal with the undesirable influence of negative emotions, which was protective for psychological state. As an important protective factor for individuals’ internal mental health, psychological capital helps individuals avoid or mitigate the negative impact of the epidemic. Individuals rely on their positive psychological expectations for the development of the epidemic and their ability to quickly recover from adversity, which is held and exercised in adverse environments, as effective resources for individuals to mitigate or reduce the impact of negative events on their mental health [45]. Individuals with rich internal positive psychological resources can alleviate and balance the psychological pressure caused by the epidemic, affect their psychological status, coping styles, and behavioral choices during the epidemic, stimulate their confidence in coping with the epidemic under stress, and reduce or mitigate the impact of the epidemic on their core belief system [46]. That is, individuals with higher psychological capital levels may regard interpersonal sensitivity as controllable and are able to recover quickly and effectively from negative emotions [47]. College students with a high level of psychological capital might rebound from interpersonal sensitivity and reduce the occurrence of anxiety symptoms through positive psychological capacity when facing the campus lockdown management.

Nevertheless, psychological capital did not moderate the path between interpersonal sensitivity and depression, inconsistent with previous findings [29]. Previous research has found that psychological capital negatively moderated the positive relationship between interpersonal sensitivity and depression on college freshmen. Empirical evidence [48] has shown that higher policy stringency might lead to poor mental health outcomes. In terms of specific policies, those that primarily kept people socially disconnected (e.g., restricting gatherings) were benefit for people body health, while were associated with more psychological problems and lower life satisfaction [49]. Furthermore, psychological capital is significantly positively associated with life satisfaction [50]. Social distancing was more strongly associated with poorer mental health assessment outcomes than campus lockdown and cancellations of public events [49]. Following a similar process, psychological capital might deplete resources, leading to unsatisfactory performance from moderating the relationship between interpersonal sensitivity and depression.

In addition, our study further revealed that psychological capital positively moderated the connection between anxiety and depression. During the period of closing campuses, college students with higher levels of interpersonal sensitivity were more likely to experience ostracism by classmates and roommates. However, previous study had found that ostracism threatened an individual’s fundamental mental requirements, including self-efficacy, self-esteem, control, and meaningful existence, which in turn damages an individual’s psychological capital [51]. Besides, because of college students have been in closed-campus for a long time, individuals with a low level of psychological needs often cannot acquire enough happiness or effective self-regulation [52]. Based on the emotional motivation theory [53], unmet mental needs will lead to anxiety, depression and other negative emotional states. Furthermore, in Chinese culture, suppressing emotions is thought a generally accepted cultural norm rather than proactive problem solving [54]. These possible explanations can be given for the moderating effect of psychological capital between anxiety and depression. Therefore, the establishment of psychological capital courses in the college curriculum system can use various educational resources to actively develop students’ psychological capital.

### Limitations

Some limitations should be taken into account when demonstrating the results of the research. First, this study was cross-sectional and cannot infer cause-and-effect relationships. Second, the data were obtained through self-report surveys, so there is subjective bias and there may be uncertainty about respondents’ memories. Third, it is possible that interpersonal sensitivity is a personality characteristic associated with psychopathology. According to the interpersonal theory of psychiatry, mental illness is mainly caused by interpersonal difficulties. Therefore, attention should be paid to the role of the special interpersonal sensitivity of college students in causing psychological adverse symptoms [55, 56]. Fourth, the bidirectional effects of interpersonal sensitivity and depression, anxiety and depression, etc., have not been thoroughly explored in this study. Finally, the conclusions result from Chinese college students, so other populations may not be generated. Future research may have respondents from diverse cultures as research subjects.

## Conclusion

In summary, the current study investigated the possible association and underlying mechanisms between interpersonal sensitivity and depression during the campus lockdown in China, and explained the mediation role of anxiety and the moderation role of psychological capital in the relation between interpersonal sensitivity and depression. It also found that the indirect effect of interpersonal sensitivity on depression decreased as psychological capital increased. The findings suggested that strict monitoring anxiety and promoting psychological capital may decrease the risk of depression among Chinese college students during the campus lockdown.

## Abbreviations

SCL-90: Symptom Checklist 90
GAD-7: Generalized Anxiety Disorder Scale
PCQ-24: Psychological Capital Questionnaire
PHQ-9: Patient Health Questionnaire

## Declarations

### Availability of data and material

The data used in this study is available from the corresponding author upon reasonable request.
